# A retrospective controlled study of postoperative fever after posterior lumbar interbody fusion due to degenerative lumbar disease

**DOI:** 10.1097/MD.0000000000029231

**Published:** 2022-05-27

**Authors:** Jung Jae Lee, Jeong Hee Kim, Ju Hee Jeon, Myeong Jong Kim, Byong Gon Park, Sang Ku Jung, Sang Ryong Jeon, Sung Woo Roh, Jin Hoon Park

**Affiliations:** a Department of Neurosurgery, Gangneung Asan Hospital, University of Ulsan College of Medicine, Gangneung, Korea; b Department of Neurosurgery, Asan Medical Center, University of Ulsan College of Medicine, Seoul, Korea; c Department of Neurosurgery, Seongnam Citizens Medical Center, Seongnam, Korea; d Department of Physiology, College of Medicine, Catholic Kwandong University, Gangneung-si, Gangwon-do 270-701, Republic of Korea.; e Department of Emergency Medicine, Gangneung Asan Hospital, University of Ulsan College of Medicine, Gangneung, Korea.

**Keywords:** diskectomy, fever, postoperative fever, spinal fusion

## Abstract

**Background:**

Postoperative fever is a common feature of spinal surgery. When fever occurs postoperatively in patients, surgeons are eager to rule out an infection. There are many reports about postoperative fever and infection; however, only a few have described the relationship between degenerative spinal disease and postoperative fever. This study aimed to investigate the causes of postoperative fever in patients with degenerative lumbar disease undergoing posterior screw fixation and interbody fusion and compare patients with non-pathologic fever and infected febrile patients.

**Methods:**

From March 2015 to February 2016, 263 patients with degenerative lumbar disease underwent posterior lumbar screw fixation and interbody fusion surgery in our institution. We performed risk factor analysis by categorizing patients as afebrile and febrile. Comparisons were made between afebrile patients and patients with non-pathologic fever, and an analysis was performed between patients with non-pathologic fever and patients with febrile infection. We compared each group by examining the demographic factors before surgery, surgery features, drain duration, and postoperative transfusion. The postoperative day (POD) of fever onset, postoperative fever duration, and blood sample results in patients with fever were investigated.

**Results:**

The drain duration was found to be an important factor between the afebrile febrile groups and between the non-pathologic fever and afebrile groups. POD of fever occurred earlier in the non-pathologic group than in the infection group (p = 0.04), and the duration of fever was shorter in the non-pathologic fever group than in the infection group (p = 0.01). Higher procalcitonin levels were observed at POD 5 in the infection group than in the non-pathologic fever group. (p < 0.01) The accidental dural rupture rate was higher in the infected group (p = 0.02); this was thought to be caused by the long non-ambulatory period after surgery.

**Conclusion:**

This study identified risk factors and differences between infectious diseases associated with postoperative fever. A significant risk factor for postoperative non-pathological fever was a shorter catheter drainage period. Fever after 3 days, fever for more than 4 days and higher procalcitonin levels after surgery suggest infection.

## 1 Introduction

Postoperative fever is a common feature of spinal surgery and has an incidence rate of 14%–91% depending on the definition of fever and the studied patient population.^[1–6]^ Usually, when fever occurs postoperatively in patients, surgeons are eager to rule out an infectious status. However, in most studies, the incidence of infection in patients with postoperative fever is less than 10%, suggesting that fever may not be a specific indicator of infection in the postoperative period.^[7,8]^ Many non-infectious factors commonly contribute to fever in patients that have undergone surgery, including the normal inflammatory cytokine response to the trauma of surgery, transfusion, perioperative medications, and hematoma.^[9–12]^ There have been many reports about postoperative fever and infection; however, only a few reports have described the relationship between degenerative spinal disease and postoperative fever. This study aimed to investigate the risk factors for postoperative fever in patients with degenerative lumbar disease who underwent posterior screw fixation and interbody fusion and compare patients with non-pathologic fever and febrile infections.

## 2 Methods

From March 2015 to February 2016, 263 patients with degenerative lumbar diseases, including lumbar stenosis and spondylolisthesis, underwent posterior lumbar screw fixation and fusion surgery at our center. Patients with a cervical or thoracic degenerative disease, spinal cord tumor, infection, or trauma were excluded from this study. Postoperative fever was explained by an increase in body temperature above 38°C.^[7]^ The body temperature of the patients was checked at least three times a day (taken by the axillary route). Patients who underwent surgery were administered intravenous prophylactic cefazolin 1 g twice daily until postoperative day (POD) 1. No patient received additional antibiotics unless the postoperative fever focus could not be detected. All patients took tridol for analgesia after surgery, and other drugs, such as non-steroidal anti-inflammatory drugs or Acetaminophen, were not taken until the onset of fever. Furthermore, for all patients, laboratory analysis of blood samples containing white blood cells (WBC), C-reactive proteins (CRP), and procalcitonin was performed on the 2nd, 5th, and 7th days after surgery to identify infected patients.

**Figure 1:**
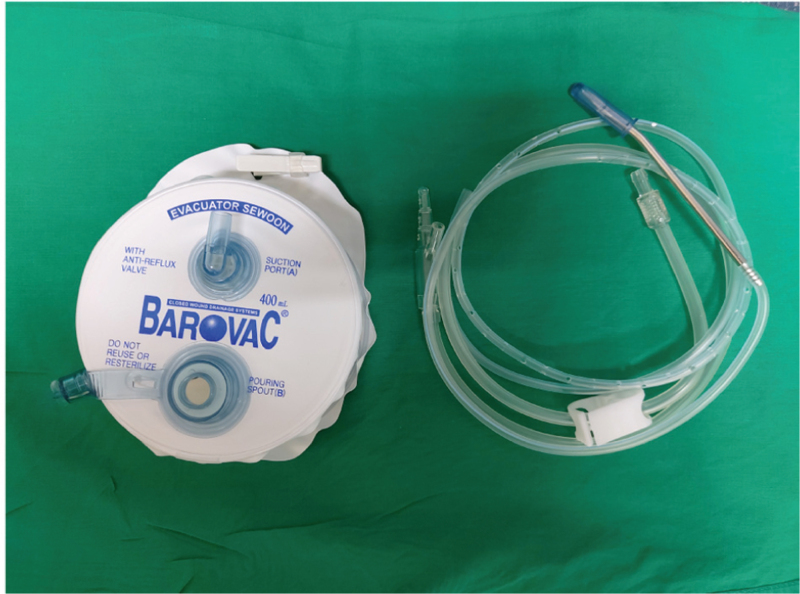
Drain product during surgery (BaroVac 400 ml, Sewoon Medical, Korea).

We evaluated the results of blood and sputum cultures, urine cultures, and wound infections to determine the presence of infection in febrile patients. When an infectious fever was suspected, an infectious disease specialist was consulted. Surgical site drains were left in all patients, and all drains used the same product (BaroVac 400 ml, Sewoon Medical, Korea) (Fig. 1). All drains were removed only when the amount of drained fluid was < 100 cc per day or > 4 days postoperatively. After surgery, all patients woke up on POD 1 but were maintained in bed rest for 5 days to prevent cerebrospinal fluid (CSF) leakage due to an accidental dural rupture during surgery. During the non-ambulatory period, the patients wore elastic stockings to prevent thromboembolism-related complications.

We performed risk factor analysis by dividing the groups into afebrile and febrile patients. In addition, to determine the risk factors of fever more precisely, comparisons were made between patients without fever and patients with non-pathogenic fever, and an analysis was performed between patients with non-pathogenic fever and those who had fever with infection.

Patient data were collected using a chart review, which included age, sex, body mass index (BMI), smoking history, bone mass density (BMD), diabetes mellitus comorbidity, duration of hospital stay, history of steroid use, operative time, surgical level, number of defecation days, drainage duration, incidental durotomy, and the presence of transfusion during the perioperative period. We analyzed the risk factors for postoperative fever using the aforementioned variables. We also checked the POD of fever and the duration of fever.

Data were presented as the mean ± standard deviation for continuous variables and as absolute or relative frequencies for categorical variables. Unpaired Student's t-tests or Mann-Whitney U tests were used to compare continuous variables, and chi-square tests or Fisher's exact tests were used for categorical variables. Statistical significance was set at p ≤ 0.05. All statistical analyses were performed using SPSS for Windows (version 14.0; SPSS Inc., Chicago, IL, USA). All data were obtained from hospital charts and imaging study databases. The study was approved by the Asan Medical Center Institutional Review Board.

## 3 Results

Of the 263 patients who met the inclusion criteria, postoperative fever was noted in 153 (58.2%). Infection was observed in 16 (10.4%) of the 153 patients with fever.

### 3.1 Risk factor analysis of postoperative fever

The differences in preoperative factors for the development of postoperative fever between afebrile and febrile patients were not statistically significant, and there were no factors to predict if the fever would occur postoperatively. However, the catheter drainage period was significantly longer in the afebrile patient group than in the febrile group (p = 0.04) (Table 1).

**Table 1:** Risk factor analysis between afebrile and febrile patient groups.

	Afebrile (N = 110)	Fever (N = 153)	*P* value
Age (years) ± SD	64.95 ± 8.81	66.23 ± 8.27	0.34
Sex (male:female)	33:77	44:109	0.82
BMI (kg/m^2^)	25.28 ± 3.36	25.59 ± 3.54	0.50
Smokers (%)	10 (9.1)	22 (14.4)	0.19
BMD (T-score)	-0.692 ± 1.863	-1.047 ± 1.798	0.14
DM (%)	25 (22.7)	33 (21.6)	0.82
Hospital stay (days)	11.60 ± 6.30	12.05 ± 5.67	0.06
Steroid users (%)	8 (7.3)	7 (4.6)	0.35
IIOP time (min)	327.33 ± 91.73	341.26 ± 93.95	0.18
Surgical level	2.23 ± 1.23	2.11 ± 1.06	0.40
No defecation (days)	2.73 ± 1.34	2.95 ± 1.27	0.18
Drainage duration (days)	3.84 ± 0.82	3.64 ± 0.79	0.04
Incidental dural tears (%)	3 (2.7)	5 (3.3)	0.80
Transfusion (%)	44 (40.0)	78 (51.0)	0.07

BMD = bone marrow density, BMI = body mass index, DM = diabetes mellitus. IIOP = operation, SD = standard deviation.

### 3.2 Analysis of risk factors between afebrile and non-pathologic fever patients

Postoperative infection did not occur in 247 (93.9%) patients. Of these non-infectious patients, fever developed in 137 patients (52.0%). The differences in the afebrile and non-pathologic fever groups were not statistically significant, except for drainage duration. The duration of the drainage catheter was longer in the afebrile group (3.84 ± 0.82 days) than in the non-pathologic group (3.66 ± 0.83 days), and this was statistically significant (p = 0.04) (Table 2).

**Table 2:** Comparison of afebrile and non-pathologic febrile groups among patients without infection.

	Afebrile (N = 110)	Non-pathologic Fever (N = 137)	*P* Value
Age (years) ± SD	64.95 ± 8.81	66.10 ± 8.35	0.41
Sex (male:female)	33:77	38:99	0.76
BMI (kg/m^2^)	25.28 ± 3.36	25.55 ± 3.57	0.57
Smokers (%)	10 (9.1)	21 (14.5)	0.19
BMD (T-score)	-0.692 ± 1.863	-1.144 ± 1.712	0.07
DM (%)	25 (22.7)	32 (22.1)	0.90
Hospital stay (days)	11.60 ± 6.30	11.87 ± 5.38	0.11
Steroid users (%)	8 (7.3)	7 (4.8)	0.41
IIOP time (min)	327.33 ± 91.73	340.47 ± 94.17	0.21
Surgical level	2.23 ± 1.23	2.10 ± 1.08	0.39
No defecation (days)	2.73 ± 1.34	2.94 ± 1.28	0.22
Drainage duration (days)	3.84 ± 0.82	3.64 ± 0.76	0.04
Incidental dural tears (%)	3 (2.7)	5 (3.4)	0.745
Transfusion (%)	44 (40.0)	74 (51.0)	0.154

BMD = bone marrow density, BMI = body mass index, DM = diabetes mellitus, IIOP = operation, SD = standard deviation.

### 3.3 Differentiation factors of infectious fever from non-pathologic fever

Of the 153 patients who had postoperative fever, 16 (10.4%) had infectious causes, including urinary tract infection (n = 8), upper respiratory infection (n = 3), pneumonia (n = 3), and wound infection (n = 2).

The rate of accidental dural rupture was higher in the infected group, but no signs of infection due to meningitis or CSF leakage were observed (p = 0.02) (Table 3).

**Table 3:** Comparison between non-pathologic febrile and febrile infected group.

	Non-pathologic Fever (N = 137)	Infection (N = 16)	*P* Value
Age (years) ± SD	66.10 ± 8.35	68.62 ± 6.54	0.42
Sex (male:female)	38:99	6:10	0.69
BMI (kg/m^2^)	25.55 ± 3.57	26.32 ± 3.14	0.47
Smokers (%)	21 (10.2)	1 (6.2)	0.33
BMD (T-score)	-1.144 ± 1.712	0.728 ± 2.504	0.02
DM (%)	29 (21.1)	4 (25.0)	0.46
Hospital stay (days)	11.87 ± 5.38	15.37 ± 9.41	0.080
Steroid users (%)	6 (4.3)	1 (6.2)	0.73
Surgery time (min)	340.47 ± 94.17	355.50 ± 94.83	0.70
Surgical level	2.10 ± 1.08	2.18 ± 0.91	0.78
No defecation (days)	2.94 ± 1.28	3.06 ± 1.23	0.72
Drainage duration (days)	3.64 ± 0.76	3.50 ± 0.89	0.49
Incidental dural tears (%)	3 (2.1)	2 (12.5)	0.02
Transfusion (%)	71 (49.0)	7 (87.5)	0.06
Fever occurence (IIPOD)	2.16 ± 0.84	2.62 ± 1.20	0.04
Duration of fever (days)	2.81 ± 1.81	4.68 ± 1.70	0.01

BMD = bone marrow density, BMI = body mass index, DM = diabetes mellitus, IIPOD = postoperative day, SD = standard deviation.

**Figure 2:**
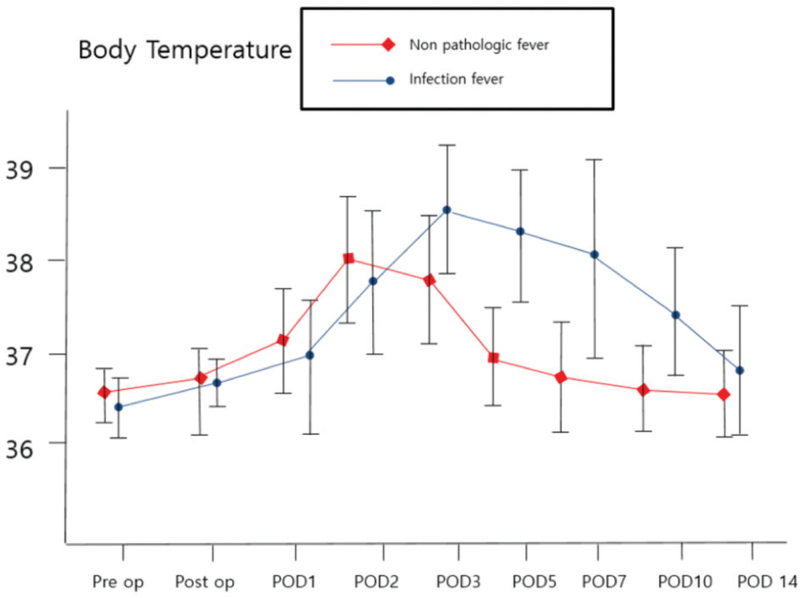
Comparison of fever patterns between the non-pathological fever group and the infected patient group according to the postoperative date (POD).

A different fever pattern was observed between the infection and non-pathologic fever groups. It was found that the POD of fever occurred earlier in the non-pathologic group than in the infection group (p = 0.04). Moreover, it was confirmed that the duration of fever was significantly shorter in the non-pathologic fever group than in the infection group (p = 0.01) (Table 3, Fig. 2).

### 3.4 Blood laboratory analyses results in patients with fever

**Figure 3:**
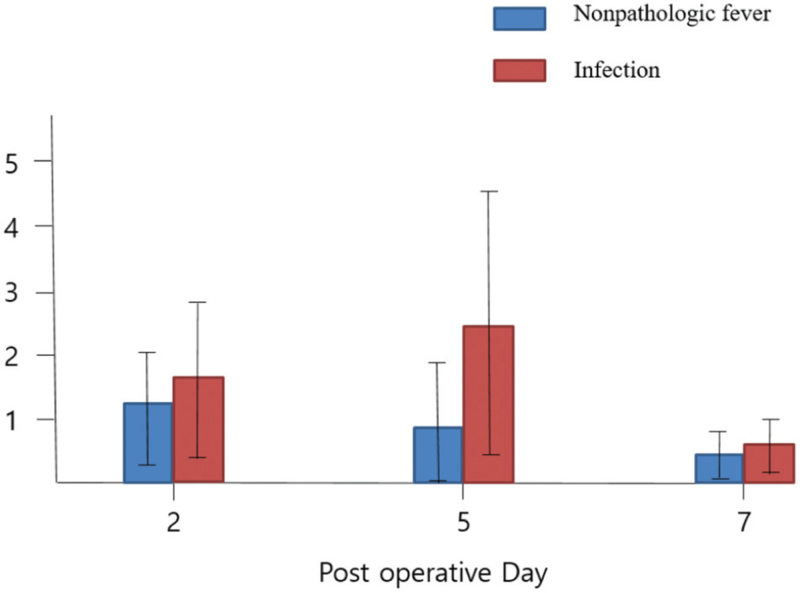
Postoperative procalcitonin analysis between infectious and non-pathologic patients among patients complaining of fever.

Blood samples from 156 patients complaining of fever were analyzed. The results show no significant differences in WBC and CRP after surgery but significant differences in procalcitonin 5 days after surgery between the infected and non-infected group (Table 4, Fig. 3).

**Table 4:** Analysis of blood samples from patients complaining of fever.

	Nonpathologic Fever (N = 137)	Infection (N = 16)	*P*-value
White Blood cell count (×10^9^ /L, mean ± SD)			
POD2	11.28 ± 2.81	11.67 ± 2.38	0.54
POD5	11.0 ± 2.59	10.9 ± 2.79	0.88
POD7	10.18 ± 2.75	9.45 ± 2.26	0.30
C reactive Protein (mg/L, mean ± SD)			
POD2	9.40 ± 3.17	9.01 ± 3.86	0.65
POD5	6.41 ± 2.36	7.06 ± 2.12	0.29
POD7	3.18 ± 1.57	3.82 ± 1.59	0.12
Procalcitonin (ng/mL, mean ± SD)			
POD2	1.22 ± 1.02	1.66 ± 1.32	0.29
POD5	0.96 ± 0.91	2.45 ± 1.93	<0.01
POD7	0.35 ± 0.34	0.54 ± 0.41	0.75

POD = Postoperative day.

## 4 Discussion

Postoperative fever commonly occurs during posterior spinal surgery. If the patient has a postoperative fever, the cause of the fever should be identified quickly, and, in particular, infectious diseases should be suspected. However, postoperative fever is not associated with infection in most cases, and overvaluation due to fever is unnecessary.^[7,8]^ Nevertheless, it is very important to quickly differentiate between infection and no infection in patients with fever after surgery. Only a few reports have investigated the relationship between degenerative spinal disease and postoperative fever.

In this study, 263 patients were retrospectively analyzed. Based on the results, there were no factors associated with postoperative fever or infection before surgery. A drain catheter was inserted to prevent postoperative hematoma. There was a significant difference in the catheter drainage duration between the postoperative febrile and afebrile groups. There was also a significant difference in the catheter drainage duration between the postoperative non-pathologic fever and the afebrile groups. Early removal of the drainage catheter may result in the accumulation of seroma or hematoma at the surgical site. The reabsorption of the seroma may cause inflammation, resulting in postoperative non-pathological fever. Previous studies have also revealed a greater prevalence of postoperative fever in patients who underwent longer surgery or traumatic surgery due to greater tissue inflammation.^[2,13]^ Seo et al. demonstrated that patients with postoperative non-pathologic fever who underwent general spinal surgery were strongly associated with surgical site inflammation induced by seroma after drain catheter removal during the late acute and subacute postoperative periods.^[8]^ Therefore, reducing the dead space on the surgical site as much as possible and meticulous bleeding control may prevent postoperative non-pathologic fever.

In our study, fever pattern was very important in determining whether the postoperative fever was due to an infection or a non-pathologic fever after degenerative lumbar spinal surgery. Factors significantly different in the two groups were the POD of fever occurrence and duration of fever, which appeared later and longer in the infected group. Thus, the later the fever develops and the longer the febrile period, the more likely it is that the cause of the fever is an infection. Based on the results of this study, if a fever occurs after POD 3 and persists for more than 4 days postoperatively, there is a possibility of infection (Fig. 2). This was an important indicator to distinguish between infection and no infection in the early postoperative stage in this study. Postoperative fever, especially during the first 48 h, is generally considered benign and self-limiting.^[7]^ However, other reports^[8,14]^ have stated that non-infectious fever may continue to occur for 3 days postoperatively and that it is difficult to differentiate infection from a normal postoperative systemic inflammatory response until 7 days postoperatively. Ward et al. suggested that postoperative temperatures above 39°C and multiple fever spikes are more likely to have an infectious origin.^[15]^ In this study, some patients in the infectious patient group also had a multiple spike pattern and a body temperature of 39°C or higher. However, other studies have demonstrated that body temperature greater than 39°C and multiple spikes should not be used as predictors of fever after infectious surgery.^[5]^ Unfortunately, the number of infected patients in this study was so small that it was difficult to make an accurate analysis of the results. This will require further research in the future.

In blood sample analysis, there was no difference in procalcitonin between the infected and non-infected subjects on the 2nd and 7th days after surgery. However, there was a significant difference in procalcitonin between the two groups on the 5th postoperative day (p < 0.01) (Table 4 and Fig. 3).

Procalcitonin is a known biomarker closely associated with bacterial infection and also correlates with its severity.^[16]^ However, previous studies found no significant increases in procalcitonin levels in viral infections or other conditions, such as SIRS (systemic inflammatory response syndrome)^[17]^ and increased procalcitonin levels were found in non-infectious conditions such as major surgery, severe trauma, or burns.^[18]^ Therefore, the presence of infectious disease must be confirmed by considering various characteristics such as the aspect of fever and the procalcitonin level.

In our study, 5 out of 8 patients with incidental durotomy complained of fever, and 2 of these patients developed an infection. Two patients who developed this infection complained of UTI and surgical wound infection, respectively (Table 5). Incidental durotomy patients had a non-ambulatory period of 5 days with a urinary catheter insertion due to the fear of CSF leakage. According to Daniel et al.,^[19]^ non-ambulatory status is a risk factor for major postoperative complications, including infections. Maintaining a urinary catheter during the non-ambulatory period is thought to induce UTI. The relationship between urinary tract infection and urinary catheterization has been well elucidated in previous studies,^[20,21]^ Among 3 patients with non-pathologic fever, 1 patient complained of atelectasis (Table 5). Early movement reduces lung complications such as atelectasis and improves lung function.^[22–24]^

**Table 5:** Characteristics of patients with incidental durotomy.

Patient No.	Age/Sex	*Underlying disease*	Drainage duration (days)	Fever duration (days)	Fever (onset)	Other complications	Infection
1	71/F	(-)	4	(-)	(-)	(-)	(-)
2	74/F	(-)	5	(-)	(-)	(-)	(-)
3	78/F	(-)	3	(-)	(-)	(-)	(-)
4	77/M	(-)	4	3	1	Atelectasis	(-)
5	82/F	DM(+)	2	3	1	(-)	(-)
6	54/F	(-)	2	5	2	(-)	(-)
7	58/M	DM(+)	3	3	2	(-)	∗UTI
8	73/F	(-)	4	4	6	(-)	Wound infection

DM = diabetes mellitus, UTI = urinary tract infection.

This study had several limitations, including those inherent to any single-institution retrospective review. Concurrent use of perioperative antibiotics, which may affect the culture results, was not analyzed. Additional factors not collected or not analyzed, including instrumentation, bone grafting, blood loss, and other intraoperative events, might have influenced the outcomes. As this was a retrospective study, the abovementioned factors could not be used as study variables because of the inaccurate recording and the use of too many kinds of instruments. Therefore, these factors were not included in the analyses. A more accurate analysis would be expected if a prospective study was conducted.

## 5 Conclusion

In conclusion, postoperative fever may be common in patients with degenerative lumbar disease undergoing posterior screw fixation and interbody fusion. This study identified the risk factors and differences between infectious diseases of postoperative fever. A shorter catheter drainage period is an important risk factor for postoperative non-pathological fever. If there is fever after 3 days and for more than 4 days, an infection can be suspected. It was also found that the longer the non-ambulatory period, the longer the urinary catheterization period, and the higher the procalcitonin level observed after surgery, the higher the possibility of infectious fever.

## Acknowledgments

We thank Byong Gon Park for assistance with the statistical analyses and Jung Jae Lee for proofreading the manuscript.

## Author contributions

**Conceptualization:** Jin Hoon Park, Ju Hee Jeon, Myeong jong Kim, Sang Ryong Jeon, Sung Woo Roh.

**Data curation:** Byong Gon Park, Jin Hoon Park, Ju Hee Jeon, JungJae Lee, Myeong jong Kim, Sang ku Jung.

**Formal analysis:** Byong Gon Park, Jin Hoon Park, Sang ku Jung.

**Investigation:** Jin Hoon Park, Myeong jong Kim, Sang Ryong

Jeon.

**Resources:** Byong Gon Park, Jeong Hee Kim.

**Software:** Byong Gon Park, Jeong Hee Kim.

**Supervision:** Jin Hoon Park, JungJae Lee, Sung Woo Roh.

**Validation:** Jeong Hee Kim, JungJae Lee.

**Visualization:** Byong Gon Park, JungJae Lee, Sung Woo Roh.

**Writing – original draft:** Ju Hee Jeon.

**Writing – review & editing:** JungJae Lee, Sung Woo Roh.
